# External Fixation versus Unreamed Tibial Intramedullary Nailing for Open Tibial Fractures: A Meta-analysis of Randomized Controlled Trials

**DOI:** 10.1038/s41598-018-30716-y

**Published:** 2018-08-24

**Authors:** Qiang Fu, Lei Zhu, Jiajia Lu, Jun Ma, Aimin Chen

**Affiliations:** Department of Orthopaedic Trauma Surgery, Changzheng Hospital, Second Military Medical University, Shanghai, 200003 China

## Abstract

Controversy exists over whether the use of external fixation (EF) or unreamed tibial intramedullary nailing (UTN) is optimal for the treatment of open tibial fractures. The aim of this study was to compare clinical outcomes in terms of postoperative superficial and deep infection, malunion, delayed union, nonunion and hardware failure between these two treatment methods. So a systematic review and meta-analysis was performed. All available randomized controlled trials that compared the clinical results of EF to those of UTN were obtained and the reported numbers of citations for each observed item were extracted to perform data synthesis. Six published randomized controlled trials with a total of 407 cases fulfilled all inclusion criteria. Data analysis revealed that UTN reduced the incidence rates of superficial infection and malunion after fixation compared with EF. However, EF led to a significant reduction in hardware failure. For postoperative deep infection, delayed union and nonunion, the treatment effects were similar between these two groups. Therefore, we recommend UTN over EF for the management of open tibial fractures. However, patients’ postoperative weight bearing should be controlled to avoid hardware failure.

## Introduction

Open tibial fractures, usually caused by high-energy trauma, are among the most common long bone fractures^1^. Clinically, the treatment of open tibial fractures remains a major therapeutic problem for surgeons because of the poor soft tissue coverage and blood supply in the tibia, with resultant difficulties arising from infection and poor bone healing^2,3^. Both bone instability and disrupted soft tissues are a current focus of all orthopedic and plastic surgeons. In the past several decades, many methods of fixation and immobilization, for instance, reamed or unreamed tibial intramedullary nailing and external skeletal fixation, have been used to treat open tibial fractures. However, the best treatment for this type of trauma remains controversial. Unreamed tibial intramedullary nailing (UTN) and external fixation (EF), the two most commonly used methods for treating open tibial fractures, continue to be subjects of debate over which is the optimal treatment.

Before the introduction of intramedullary nailing, the most widely accepted surgical treatment for open tibial fractures was EF. This technique gained widespread popularity because of its fast and easy application without additional blood supply irritation or soft tissue stripping. However, it has several disadvantages, including pin loosening, high incidences of malunion and pin site infection, difficulties with managing the soft tissue of the wound and reliance on patient compliance for pin-track hygiene^4–8^. Intramedullary fixation, represented by unreamed tibial intramedullary nailing, is a relatively new additional treatment option for open tibial fractures. It offers stable fracture fixation with short-term immobilization, which can allow for early weight bearing. A small diameter UTN minimizes disruption to the remaining tibial intramedullary blood supply and therefore reduces complications, such as nonunion and malunion^9–12^. Better cosmesis and high patient acceptance are additional advantages over EF^13^. However, there is the potential for hardware failure and the spread of infections through the medullary canal^4,14^.

To solve this debate over which is the optimal treatment, some systematic reviews and meta-analyses have been conducted. Fang *et al*.^15^ and Zhang *et al*.^16^ conducted meta- analyses to compare the two methods for treating Gustilo III tibial fractures. However, some of the studies included in their meta-analyses were retrospective studies and case series, which could have resulted in certain biases. Xu *et al*.^17^ conducted meta-analysis of randomized clinical trials (RCTs) of the use of external fixators versus intramedullary nails to treat open tibial fracture. These authors suggested that intramedullary nails are more effective than external fixators. Unfortunately, this meta-analysis did not make a distinction between reamed and unreamed tibial intramedullary nailing, and the heterogeneity of interventions may have led to an unreliable conclusion. Considering the above limitations, we aimed to conduct a more rigorous meta-analysis of high-quality RCTs to compare EF with UTN in the treatment of open tibial fractures. We aimed to perform a scientific evaluation of all available literature to increase the current understanding of the management of this trauma.

## Materials and Methods

### Search strategy

An English language literature search of PubMed, Cochrane Library, EMBASE, BIOSIS, Ovid and the Cochrane Central Register of Controlled Trials (1980 to July 2018) was performed with the following Medical Subject Heading (MeSH) terms used in different combinations: open tibial fractures, external fixation, unreamed tibial intramedullary nailing, treatment outcome, and comparative study. To identify other relevant studies, we also reviewed the bibliographies of the identified trials and review articles. Only those with full texts available were considered.

### Study selection and outcome measures

**Figure 1:**
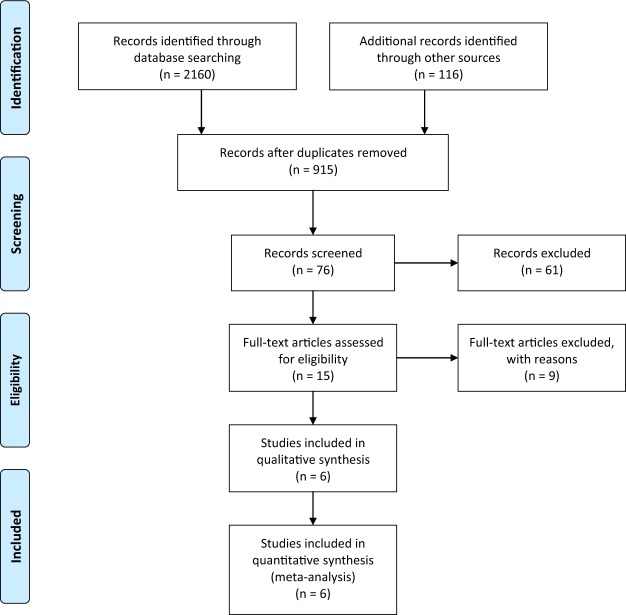
PRISMA 2009 flow diagram.

We included articles based on the following criteria: (1) the article type was strictly limited to a RCT assessing the treatment of open tibial fractures; (2) the intervention was external fixation versus unreamed intramedullary nailing; and (3) the study reported at least 1 of the following outcomes of interest: (i) postoperative superficial infection, defined as clinical findings of superficial and local erythema, swelling, and tenderness of the wound or pin track, which were resolved with the administration of antibiotics; (ii) postoperative deep infection, defined as purulent drainage or osteomyelitis presenting after definitive wound healing and diagnosed based on clinical suspicion and subsequent culturing, which required prolonged antibiotics or surgical debridement; (iii) malunion, defined as a varus or valgus angulation of more than 5 degrees, anterior or posterior angulation of more than 10 degrees, shortening of more than 1.5 cm or more than a 0.5 cm gap at the fracture site; (iv) delayed union, defined as lack of bridging callus at 5 months that eventually healed within seven to nine months; (v) nonunion, defined as absence of a bridging callus across a fracture site after the expected time interval for that injury (usually 10 months); and (vi) hardware failure, defined as pin breakage in EF cases or main nail or locking screw breakage in UTN cases. Retrospective studies, biomechanical studies, literature reviews and studies that did not provide sufficient data, such as the patients’ demographic characteristics or information regarding surgery, diagnosis, follow-up and clinical outcomes, were all excluded. A flow chart of study selection process is presented in Fig. 1.

### Data extraction and quality assessment

All eligible studies were reviewed independently by 2 reviewers, and related data were extracted using a data collection form. Extracted data included patient characteristics (sample size, mean age, and proportion of females), fracture types (Gustilo-Anderson classification^18^), protocol for the treatment of fractures, follow-up duration and the above-mentioned outcomes of interest. When relevant data were missing, we contacted the authors and asked them to supply the data. We quantified study quality using the “Cochrane Collaboration’s tool for assessing risk of bias”, which is a quality assessment tool recommended in the Cochrane Handbook (version 5.1.0, updated March 2011) that includes six major possible sources of bias: random sequence generation, allocation concealment, blinding of participants and personnel, incomplete outcome data, selective reporting and anything else^19^. A third reviewer adjudicated any disagreements about the extracted data and checked the extracted data for accuracy. The data were then checked and entered into Review Manager (Version 5.2. Copenhagen: The Nordic Cochrane Centre, The Cochrane Collaboration, 2008) database for further analysis.

### Data analysis

The clinical outcomes pooled across studies were analyzed according to the risk ratios (RRs) and corresponding 95% confidence intervals (CIs) to assess differences between the two treatment methods. Moreover, statistical heterogeneity across trials was quantified with the I^2^ statistic according to the PRISMA guidelines^20^. I^2^ values of less than 25% were considered homogeneous, and those of 25%, 50%, and 75% or more represented low, moderate, and high heterogeneity, respectively^21^. For homogeneous studies or those with low statistical heterogeneity, the fixed-effects model was used todetermine the overall RR. Otherwise, the random-effects model was used^22^. Sensitivity analyses (excluding one study at a time) were conducted to assess the heterogeneity and robustness of the pooled results. We assessed potential publication bias using a funnel plot. All tests were two-tailed, and a P value of less than 0.05 was considered significant.

## Results

### Literature search

All potentially relevant articles and abstracts were reviewed, and 6 published RCTs with a total of 407 cases (188 EF and 219 UTN) were determined to fulfill all inclusion criteria for our meta-analysis^23–28^. The included study characteristics are summarized in Table 1, which presents the author information, year of publication, sample size, patient age range, gender, interventions, follow-up duration and fracture types. No differences in sex, age or injury mechanisms were reported in any of the studies; therefore, the related baseline characteristics were considered comparable.

**Table 1:** Characteristics of included studies.

Author	Cases (n)		Gender (M/F)		Mean age (y)		Follow-up time (m)		Types of fractures (Gustilo-Anderson)	
	UTN	EF	UTN	EF	UTN	EF	UTN	EF	UTN	EF
Holbrook, 1989	29	28	/		28(15–66)	25(7–65)	16.8(14–21)	18.5(12–24)	6I/12II/10III	8I/15II/6III
Tornetta, 1994	15	14	11/4	9/5	41(21–73)	37(19–86)	21(19–36)		15IIIB	14IIIB
Tu, 1995	18	18	30/6		38.5(16–65)		20.5(18–24)		10IIIA/8IIIB	10IIIA/8IIIB
Henley, 1998	104	70	79/21	53/15	33(14–81)	33(16–77)	15.7	17.6	51II/41IIIA/12IIIB	22II/34IIIA/14IIIB
Inan, 2007	29	32	24/5	28/4	31.7(17–54)	32.3(15–64)	43.3(30–61)	46.5(33–67)	29IIIA	32IIIA
Mohseni, 2011	25	25	20/5	22/3	30.8 ± 5.24	28.92 ± 8.88	12		15IIIA/10IIIB	13IIIA/12IIIB

UTN – unreamed tibial intramedullary nails; EF – external fixator; M – male; F – female; n – number; y – year; m – month.

### Meta-analysis of postoperative infection

**Figure 2:**
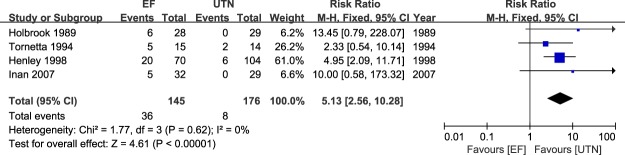
Table and forest plot illustrating the risk ratios for superficial infection for EF and UTN during the follow-up period.

**Figure 3:**
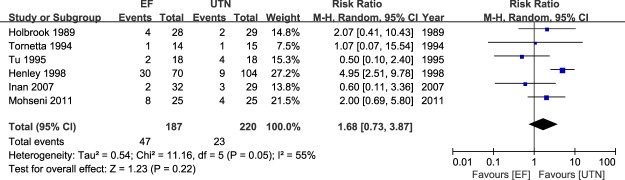
Table and forest plot illustrating the risk ratios for deep infection for EF and UTN during the follow-up period.

Postoperative infection was among the most commonly reported outcome measures in the included studies. Four studies^23,24,26,28^ independently reported the superficial infection statuses of the patients, and all six studies^23–28^ reported deep infection data. Analysis revealed a significant difference in the pooled treatment effect favoring UTN, with no heterogeneity (RR = 5.13; 95% CI: 2.56 to 10.28; P < 0.00001; I^2^ = 0%; Fig. 2). However, deep infection analysis revealed no significant difference between the two treatment arms, with moderate heterogeneity (RR = 1.68; 95% CI: 0.73 to 3.87; P < 0.22; I^2^ = 55%); therefore, no method was favored (Fig. 3).

### Meta-analysis of malunion

**Figure 4:**
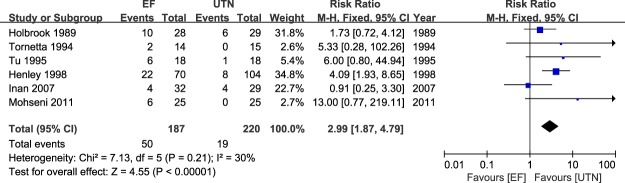
Table and forest plot illustrating the risk ratios for malunion for EF and UTN during the follow-up period.

The occurrence rates of malunion in the patients treated with EF and UTN were pooled across all six studies^23–28^. As shown in Fig. 4, significantly less malunion was observed in the patients who underwent UTN compared with those who underwent EF, with minimal heterogeneity (RR = 2.99; 95% CI: 1.87 to 4.79; P < 0.00001; I^2^ = 30%).

### Meta-analysis of delayed union and nonunion

**Figure 5:**
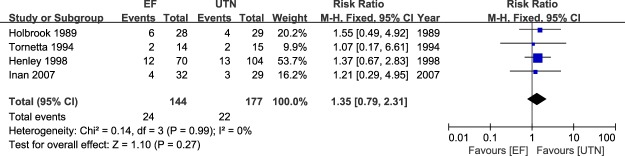
Table and forest plot illustrating the risk ratios for delayed union for EF and UTN during the follow-up period.

**Figure 6:**
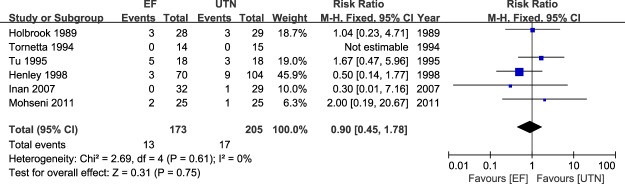
Table and forest plot illustrating the risk ratios for nonunion for EF and UTN during the follow-up period.

The clinical outcome of delayed union was evaluated in four studies^23,24,26,28^. Meta-analysis revealed no significant difference between the two methods, with no heterogeneity (RR = 1.35; 95% CI: 0.79 to 2.31; P = 0.27; I^2^ = 0%; Fig. 5). In addition, all six studies^23–28^ provided the incidence rates of nonunion for the two treatment arms. As shown in Fig. 6, there were no significant differences in the incidence of nonunion, with no evidence of statistical heterogeneity (RR = 0.90; 95% CI: 0.45 to 1.78; P = 0.75; I^2^ = 0%; Fig. 6). Therefore, for the parameters of delayed union and nonunion, no method was favored.

### Meta-analysis of hardware failure

**Figure 7:**
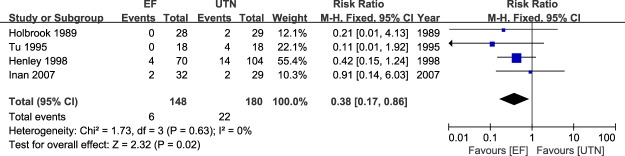
Table and forest plot illustrating the risk ratios for hardware failure for EF and UTN during the follow-up period.

The data related to hardware failure were pooled across four studies^23,24,27,28^. The meta-analysis results are presented in Fig. 7. The EF group showed a significantly lower incidence of hardware failure after fixation compared with the UTN group, with no heterogeneity (RR = 0.90; 95% CI: 0.45 to 1.78; P = 0.75; I^2^ = 0%).

### Sensitivity and publication bias analyses

To investigate the potential publication bias, funnel plots were generated based on the results of malunion analysis. The plots did not reveal obvious asymmetry, suggesting that publication bias may not play an important role in the observed effect. The robustness of the results was assessed by sensitivity analyses, which demonstrated that no individual study predominantly affected the overall RR.

## Discussion

The universally accepted protocols for managing open tibial fractures include immediate debridement and irrigation, administration of antibiotics, skeletal stabilization, delayed wound closure and early soft tissue coverage^29,30^. However, there is no consensus on the best method of bony stabilization. EF and UTN are two well-accepted techniques, and they are also associated with the most controversy over which is the optimal treatment. According to the best estimates of our meta-analysis, UTN reduced the incidence rates of superficial infection and malunion after fixation compared with EF. However, EF had a significantly lower rate of hardware failure. For postoperative deep infection, delayed union and nonunion, the treatment effects were similar between the two groups.

Meta-analysis of RCTs is generally considered to provide the highest evidence of clinical interventions, with advantages over observational research studies and single randomized trials. In our meta-analysis, we performed explicit literature searches and selected the only six eligible RCTs. We also used the Cochrane risk-of-bias tool to assess the quality of evidence for the main outcomes. In addition, the funnel plots and sensitivity analysis indicated that the meta-analysis results were robust and reliable. Thus, we have reason to believe that our results are valid.

One of the basic goals in the treatment of open tibial fractures is to prevent infection. Deep infection is an important factor for predicting patient prognosis in terms of limb salvage and preservation of function^31^. Previous studies have indicated that UTN may increase the risk of infections in the medullary canal and even the risk of amputation^4,14^. However, in our meta-analysis, no significant difference in the rates of deep infection was detected between the two groups. Kaftandziev *et al*.^32^ have noted that sterile metal does not cause infection but that sterile metal combined with inadequate debridement or absent soft tissue coverage does lead to infection in devitalized soft tissue and bone^32^. An important factor in the body’s ability to resist infection is the viability of surrounding soft tissue. Consequently, adequate debridement and early soft tissue coverage may be the keys to preventing deep infections and producing favorable results.

Superficial infection often manifests as pin-site infection in EF and incision infection in UTN. Our meta-analysis revealed a higher incidence of superficial infection in the EF patients, which is consistent with previously published work^33,34^. In our opinion, thorough debridement, rigorous wound management and reasonable antibiotic usage can reduce the risk of superficial infections to a lower and more acceptable level.

The incidence of malunion was investigated and was found to be higher in EF, in accordance with some other meta-analyses^15–17^. Some published studies have reported that open tibial fracture patients treated with EF may experience malunion, with an incidence of up to 20%^5,35^. On the one hand, when external fixation is used, accurate initial anatomic restoration with a limited exposure of the wound site is relatively difficult. On the other hand, the external fixation moment arm is much higher, which gives substandard alignment control mechanically. In contrast, the intramedullary nail is much closer to the fracture site. It can provide robust stability and therefore effectively maintain alignment, which could contribute to the lower incidence of malunion. However, healing bone cannot distinguish between correct and incorrect alignment patterns. Once the healing process begins, final bone healing can be achieved in both groups despite the alignment condition^15^. As a result, the differences in delayed union and nonunion were not significant between these two groups in this meta-analysis.

Hardware failure remains the most reported complication of UTN, with an incidence of up to 3–16%^36,37^. The most common hardware failure is the breakage of locking screws. Among 22 cases of UTN hardware failure reported in our study, 19 cases were locking screw breaks and only 3 main nail breaks were found. In a prospective study conducted by Court-Brown^38^, the screw breakage rate was found to be as high as 52% when UTN was used. However, Alberts *et al*.’s research^39^ showed that locking screw failure’s long-term effect is minor because in most cases this complication could not be noticed in the first eight weeks and did not result in more than 5 mm of shortening. Generally speaking, these failures are related to fracture patterns, fracture locations and the patients’ weight bearing statuses. Unlike the compound system of nail and bone in reamed nails, UTN functions as a splint in the medullary cavity. The load is transmitted directly to the locking screws^36^. Awareness of this function and adherence to a strict protocol concerning patient mobilization and weight-bearing status appear to be the most important factors for avoiding this kind of failure.

The functional outcome is also a focus after fracture surgery. An obvious advantage of UTN is that is can allow for early range of motion after surgical intervention. In contrast, for the EF technique, the passing of wires and pins through muscles may limit motion and joint contracture^40,41^. Some patients are not able to perform functional exercises until the external fixator is removed. Unfortunately, the included RCTs did not use systematic scores or validated standards to measure lower extremity functional outcomes, and we were unable to obtain quantitative proof from the included references to complete data analysis. Therefore, additional RCTs should be performed to improve the objective indices of the postoperative functional assessment.

Our meta-analysis has some limitations. The first is the lack of allocation concealment measures in the included studies. However, we believe that this limitation did not substantially affect the treatment outcome. Moreover, we did not evaluate treatment cost, which is also a subject of current debate. Finally, the surgeons’ different levels of surgical experience could also have influenced the results. For example, UTN is a more complicated technique than EF. To avoid these limitations, more RCTs with higher methodological quality are needed to produce more convincing evidence.

## Conclusion

The results of this meta-analysis revealed that UTN significantly reduced the incidence rates of superficial infection and malunion compared with EF, suggesting that it is likely a safe and effective alternative to EF for treating open tibial fractures. Therefore, we recommend UTN over EF for the management of open tibial fractures. However, patients’ postoperative weight bearing should be controlled to avoid hardware failure.
